# Improving the Quality of Bio-oil Using the Interaction of Plastics and Biomass through Copyrolysis Coupled with Nonthermal Plasma Processing

**DOI:** 10.1021/acs.energyfuels.3c04082

**Published:** 2023-12-29

**Authors:** Maryam Khatibi, Mohamad A. Nahil, Paul T. Williams

**Affiliations:** School of Chemical & Process Engineering, University of Leeds, Leeds LS2 9JT, U.K.

## Abstract

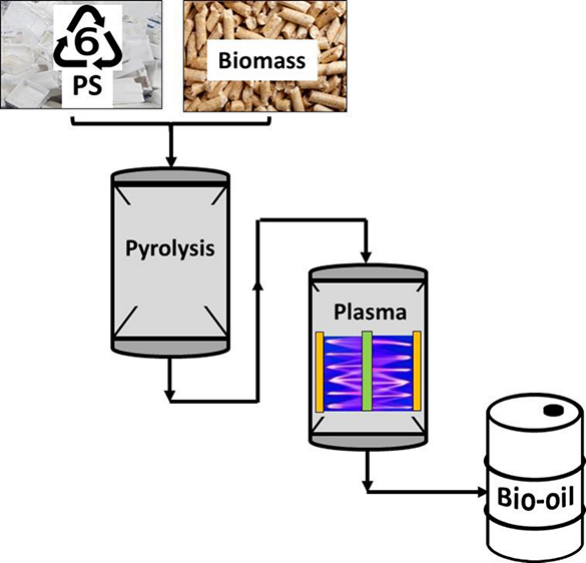

Bio-oil produced from the pyrolysis of biomass is chemically complex, viscous, highly acidic, and highly oxygenated. Copyrolysis of biomass and plastics can enhance oil quality by raising the H/C ratio, leading to improved biofuel properties. In this work, copyrolysis of polystyrene and biomass was passed to a second-stage dielectric barrier discharge nonthermal plasma reactor with the aim to further improve the product bio-oil. Pyrolysis of the polystyrene and biomass produces volatiles that pass to the second stage to undergo cracking and autohydrogenation reactions under nonthermal plasma conditions. There was a synergistic interaction between biomass and polystyrene in terms of overall oil and gas yield and composition even in the absence of the nonthermal plasma. However, the introduction of the nonthermal plasma produced a marked increase in monocyclic aromatic hydrocarbons (e.g., ethylbenzene), whereas polycyclic aromatic compounds decreased in concentration. Most notably, the influence of the plasma markedly reduced the quantity of oxygenated compounds in the product oil. It is suggested that the unique reactive environment produced by the plasma involving high-energy electrons, excited radicals, ions, and intermediates increases the interaction of the polystyrene and biomass pyrolysis volatiles. Increasing input plasma power from 50 to 70 W further enhanced the effects of the nonthermal plasma.

## 1 Introduction

Global energy demand is rapidly increasing, and fossil-based fuels including coal, oil, and natural gas are currently meeting the majority of that need with their consequent environmental and climate change impact.^1^ Given the limited supply of fossil fuels, it is critical to find alternate energy sources to meet demand.^2,3^ Biofuels derived from renewable sources reduce pollution in the atmosphere and aid in the socioeconomic development of rural communities.^4^ Biomass is attractive as a renewable energy source because it can be used to generate electricity, heat, and liquid and gaseous fuels for the industrial and transport sectors.^5^ Some of the well-known thermochemical procedures to produce bio-oil, syngas, and liquid fuels include pyrolysis, liquefaction, and gasification.^6^

Pyrolysis has been promoted as an effective method for producing liquid biofuels^7^ and can be used with a wide range of feedstocks, particularly from wastes, such as wood and agroforestry residues, making it versatile and adaptable.^8^ The product bio-oil can be viewed as a promising liquid fuel in that it has an equivalent energy value of 70–95% to that of petro-crude.^4^ However, the main drawbacks of crude bio-oils obtained from the pyrolysis of biomass wastes are poor quality due to the presence of water and oxygen components, acidic with a pH range from 3.5 to 4.2, high viscosity, thermal and chemical instability, low heating value, low calorific value, and immiscibility with hydrocarbons.^9−13^ Therefore, oxygen removal is a critical stage in biofuel upgrading.^13^

Copyrolysis of biomass and plastics, in which the plastic polymers act as hydrogen donors and therefore increase the H-transfer process and reduce oxygenated compounds in the product oil, has been proposed as a promising bio-oil upgrading process.^14,15^ Adding hydrogen to the biomass pyrolysis process via copyrolysis of a material with a high hydrogen content such as waste plastics raises the H/C ratio and changes the oxygen removal reaction mechanism by substituting decarbonylation and decarboxylation processes with dehydration reactions.^16−19^ Free radical interactions derived from the plastic polymer interact with the biomass volatile compounds contributing to the synergistic effect in the copyrolysis process.^17^ Thereby, the interaction synergistically lowers the oxygen content and increases the hydrocarbon content of the product bio-oil.

The most commonly investigated plastics for copyrolysis with biomass are polyethylene, polypropylene, and polystyrene because of their high hydrogen content.^20^ Polystyrene has been less investigated compared with polyethylene and polypropylene, but polystyrene is attractive as a feedstock for copyrolysis with biomass in that it has an aromatic polymer structure with a high hydrogen content; in addition, it is produced in large quantities as a waste plastic, of the order of ∼17 million metric tons per year.^21^ Pyrolysis of polystyrene with biomass as a copyrolysis process has been used to produce liquid fuels and to improve end-product bio-oil quality. For example, Sanahuja-Parejo et al.^15^ studied copyrolysis of biomass waste and polystyrene in a fixed-bed reactor. The addition of polystyrene (5–40 wt %) to the pyrolysis of the biomass resulted in significant favorable synergistic effects. The organic liquid fraction increased when the proportion of polystyrene was increased, reaching up to 85 wt % at the highest percentage (40 wt %). An increase in aromatic compounds and reduced concentrations of phenols were reported, with the polystyrene acting as a hydrogen donor, encouraging oligomerization, cyclation, and hydrodeoxygenation processes.

Nonthermal plasma processing has been applied in combination with pyrolysis to produce liquid fuels due to the unique nonequilibrium reaction environment of the nonthermal plasma. Dissociation, electron collision excitation, and ionization inside the nonthermal plasma yield excited molecules, atomic or molecular ions, metastable species, and neutral atoms at low temperature (<200 °C). The average temperature of electrons in the nonthermal plasma is much higher than that of the surrounding gas molecules. For example, it has been reported that the electron energy is up to 10 eV, which is equal to temperatures approaching >10,000 °C, whereas the temperature of the surrounding gas remains low.^22^ Recombination of the generated reactive chemical species produces neutral molecules with an upgraded product value.^23^ The nonthermal plasma may be generated using different devices including dielectric barrier discharge (DBD), gliding arc, and corona reactors. The DBD process is generated between two electrodes as an electric discharge with a large potential difference creating an intense electrical field and high energy plasma in the gas between the electrodes; the DBD process has a simple design and operation.^24^ In the particular application described in this work, the application of nonthermal plasma process results in the production of hydrogen radicals produced by the cracking of alkylated chemical groups associated with the pyrolysis volatile compounds found in the pyrolysis products from biomass and plastics.^25^ This nonthermal plasma, *in situ* generated hydrogen, thereby eliminates the need for an external H_2_ supply to hydrogenate the biomass pyrolysis volatiles. Furthermore, the nonthermal plasma reactor has several benefits over the catalytic process, including operation at low temperature and atmospheric pressure and reduced catalyst coke formation.^9^ Nonthermal plasma produces extremely reactive species, and the nonequilibrium features within the plasma can overcome thermodynamic barriers in chemical reactions.^26^ In comparison to conventional hydrodeoxygenation, nonthermal plasma upgrading provides additional processing advantages, including a higher conversion rate and increased formation of deoxygenated products.^27^

There are few reports investigating the copyrolysis of biomass and polystyrene coupled with nonthermal plasma processing of the evolved pyrolysis volatiles with the aim of using the plastics as a hydrogen donor to improve the quality of the derived bio-oil. Whereas the interaction of plastics and biomass has been extensively studied with a view to upgrading the product bio-oil,^28,29^ there are not many studies involving the additional enhancing effect of nonthermal plasma. There is some work on the addition of nonthermal plasma with copyrolysis of plastics and biomass,^30,31^ but it is almost always in the presence of a catalyst, which further complicates the process. Also, there are few reports on the copyrolysis of biomass with polystyrene coupled with nonthermal plasma processing to improve the characteristics of the product oil. However, copyrolysis of biomass and polystyrene (without plasma) has been shown to produce a low viscosity product oil with a higher yield than biomass alone.^15,29^ The pyrolysis of polystyrene alone produces a high yield (>90 wt %), highly aromatic liquid oil, composed of mainly the monomer styrene. Additionally, the addition of polystyrene to biomass also acts to reduce the oxygen content of the product oil through a hydrogen donor process.^29^ Knowledge of the copyrolysis of biomass and polystyrene with nonthermal plasma postprocessing of the pyrolysis volatiles, with detailed analysis of the product oils and without the complicating factor of catalyst promotion, would increase the understanding of the process.

The present study is aimed at investigating the pyrolysis of polystyrene and biomass and copyrolysis of a 1:1 mixture of biomass and polystyrene followed by upgrading of the produced pyrolysis volatiles using a two-stage pyrolysis and DBD nonthermal plasma process. The detailed chemical composition of the product oils and gases is reported in relation to the different feedstocks and at different input plasma power.

## 2 Materials and Methods

### 2.1 Materials

The feedstock used for the investigation consisted of waste biomass and waste polystyrene. The biomass was prepared to be representative of the mixture of the main biomass waste materials found in municipal solid waste and consisted of 16 wt % wood, 42 wt % newspaper, and 42 wt % cardboard. The wood was obtained as waste wood from Liverpool Wood Pellets Ltd., Liverpool, U.K. The wood was shredded and sieved to achieve the particle size of 1 mm. Newspaper and cardboard were purchased from local stores and were cut and sieved to the particle size of 1 mm. The waste polystyrene with the particle size of 1–2 mm was supplied by Regain Polymers Limited, Castleford, U.K. The proximate and ultimate (C, H, O, N, S) analyses of the wood, newspaper, cardboard, and polystyrene were determined using a Schimadzu TGA-50 thermogravimetric analyzer (TGA) and Thermos EA-2000 elemental analyzer, respectively. The results are shown in Table 1. The higher heating value (HHV) of the feedstocks was calculated using eq 1.^32^
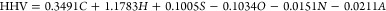
1
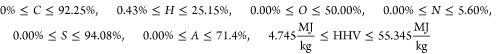


**Table 1:** Proximate and Ultimate Analyses of Polystyrene, Newspaper, Wood, and Cardboard

	**polystyrene**	**newspaper**	**wood**	**cardboard**
**proximate analysis** (wt %)				
volatile	98.6	73.3	79.4	74.1
fixed carbon	0	10.0	14.5	8.8
ash	1.2	11.5	0	11.5
moisture	0	5.3	6.1	5.7
**elemental analysis** (wt %) dry basis				
carbon	89.3	44.1	50.2	45.8
hydrogen	9.0	6.0	6.5	5.7
nitrogen	0.4	0.4	0.3	0.5
oxygen	0	37.3	43.0	35.9
HHV (MJ/kg)	41.7	18.4	20.8	18.7

In this context, *C*, *H*, *O*, *N*, *S*, and *A* show the carbon, hydrogen, oxygen, nitrogen, sulfur, and ash contents of the materials, respectively. These values are expressed as mass percentages on a dry basis.^32^

### 2.2 Experimental Reactor System

**Figure 1:**
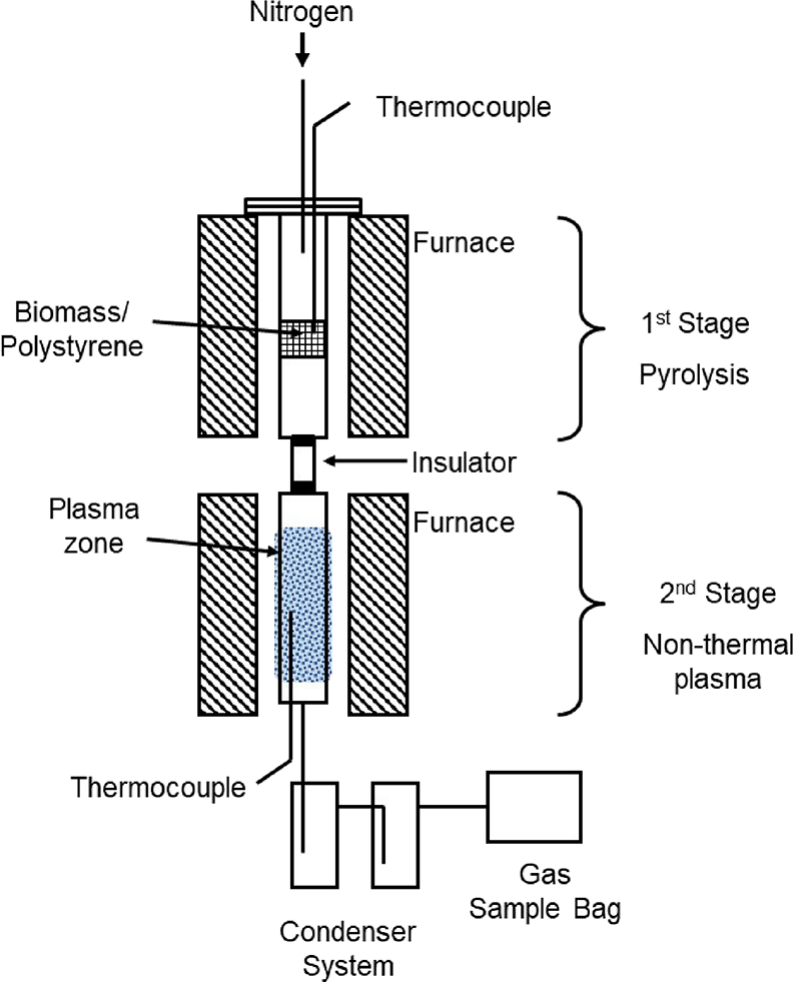
Schematic diagram of the pyrolysis-DBD plasma reactor system.

**Figure 2:**
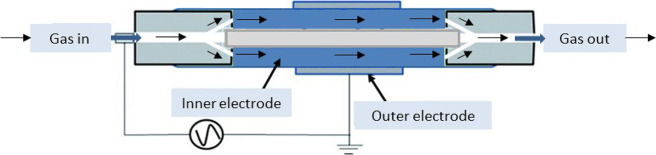
Schematic diagram of the nonthermal plasma reactor.

The pyrolysis-nonthermal plasma experiments were carried out in a two-stage reactor system as shown in Figure 1. In the first stage, pyrolysis reactions took place, decomposing the waste biomass and waste plastics, and the produced pyrolysis volatiles were passed to a second-stage DBD nonthermal plasma reactor for plasma upgrading. Nitrogen was used as the purge carrier gas. The first-stage pyrolysis reactor was a fixed-bed design, constructed of stainless steel of 250 mm length and 20 mm internal diameter. The reactor was surrounded and heated by a temperature-controlled electric tubular heating furnace. A stainless-steel crucible was hung from the reactor lid, and the feedstock was held inside the pyrolysis reactor. An electric ceramic insulator connecting tube was placed between the pyrolysis and plasma reactors. The second-stage DBD nonthermal plasma reactor was constructed of quartz glass of 23 mm diameter. The nonthermal plasma reactor was a coaxial DBD plasma reactor, which consisted of two electrodes as shown in Figure 2. The inner stainless-steel electrode was 254 mm in length and 18 mm in diameter and was connected to the power supply and located in the middle of the reactor. The second outer electrode was a copper mesh 95 mm in length, which was used as the low-voltage electrode, and it was wrapped around a quartz glass tube. The quartz tube acted as a dielectric material, separating the inner and outer electrodes. The high voltage was connected to the inner electrode, and the outer electrode was connected to a ground. The discharge zone, where plasma reactions take place, was the region between two electrodes with 95 mm axial length with a discharge gap of 5 mm. The DBD plasma reactor was supplied with an AC high-voltage power supply, frequency of 1500 Hz, and maximum peak-to-peak voltage of 20 kV. A power supply generator was used to set the process parameters, such as the frequency. When the power supply is switched on, a plasma is generated in the plasma zone, and the amount of discharge input power (50 or 70 W) is controlled manually using a voltage adjusting regulator. A digital oscilloscope monitored the discharge. The applied electric field ionizes the evolved pyrolysis gases and purge gas (N_2_) molecules in the discharge zone to generate electrons that collide with the pyrolysis derived molecular species in the discharge gap, which leads to the production of reactive components that aid in the initiation and propagation of chemical reactions. The product gases from the DBD reactor exited through an air-cooled condenser and then a dry ice-cooled condenser to condense the product liquids. Noncondensable gases were collected in a 25 L Tedlar gas sample bag.

The experimental procedure involved initial heating of the DBD nonthermal plasma reactor to the desired temperature of 250 °C. The temperature of 250 °C was chosen to prevent any condensation of liquids within the plasma reactor. The pyrolysis reactor contained 4.0 g of feedstock (polystyrene or biomass) and, for the 1:1 mixture, 2.0 g of polystyrene and 2.0 g biomass. Pyrolysis of the feedstock consisted of heating to a temperature of 650 °C with a heating rate of 20 °C min^–1^ and holding at 650 °C for 20 min. When the pyrolysis temperature reached 200 °C, the power supply to the DBD reactor was switched on to generate the plasma. The product gases were collected in the gas sample bag to be analyzed using gas chromatography (GC). The liquid was collected from the condensers using dichloromethane solvent and then analyzed by gas chromatography–mass spectrometry (GC–MS).

Products yields were calculated based on eqs 2–4. Masses of gas compounds were calculated using the ideal gas law.
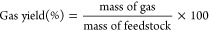
2
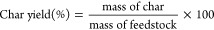
3
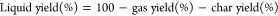
4

The experiments were repeated at least twice. To validate the experimental reactor system, many experiments were conducted at the same process conditions using a mixed biomass/plastic feedstock and demonstrated excellent experimental repeatability. For example, the relative standard deviations for the yield of gas, liquid, and char were 6.57, 6.24, and 3.02%, respectively, and for H_2_, CO, CO_2_, and CH_4_, the relative standard deviations were 0.63, 9.11, 4.71, and 6.16%, respectively.

### 2.3 Gas Analysis

The produced gases were analyzed by packed column GC using a set of Varian 3380C gas chromatographs to determine gas composition. GC-TCD was used to specify the composition of permanent gases, including H_2_, O_2_, CO, and N_2_, with argon as the carrier gas and a 60–80 mesh molecular sieve column. GC-TCD also analyzed the product gas for CO_2_ with argon as the carrier gas and a HayeSep 60–80 mesh molecular sieve as the column packing. GC-FID was applied for the determination of C_1_–C_4_ hydrocarbons using nitrogen as the carrier gas and a column packed with 80–100 mesh HayeSep.

### 2.4 Oil Analysis

The product oil trapped in the condensers was collected and analyzed for water content (for biomass-derived products) using Karl Fischer volumetric titration using a Metrohm890 Titrando apparatus and Tiamo 2.3 software. Bio-oil compositions were investigated by GC–MS with a Hewlett-Packard 5280 GC and an HP 5271 ion trap mass spectrometric detector. The GC column was a Restek RTX-5MS fused silica column of 30 m length × 0.25 mm i.d. The solid phase of the column was 95% dimethyl polysiloxane and 5% diphenyl of 25 μm film thickness. The carrier gas used for the GC–MS was helium. The oils were dissolved in dichloromethane prior to injection into the GC–MS. The GC–MS total ion chromatographic peaks in relation to their chromatographic retention times were used to identify and quantify the compounds in the oil, supported by the use of the NIST 2008 spectral library. Compounds were identified where an ion mass spectral similarity index of >70% was recorded.

Quantification of compounds was calculated using eqs 5 and 6.
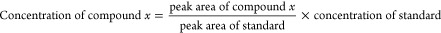
5
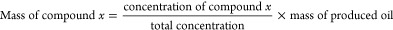
6

### 2.5 Synergistic Effect

The synergistic effect was determined where the copyrolysis product yields were compared to the theoretical value computed by the additivity rule from the yields corresponding to the individual components, as shown in eq 7.^19^
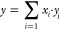
7where *y* is the theoretical value, *y_i_* is the experimental values derived from individual biomass and plastic pyrolysis, and *x_i_* is the biomass/plastic mix mass proportion. A synergistic effect occurs during copyrolysis if the experimental value exceeds the theoretical value.^19^

## 3 Results and Discussion

### 3.1 Product Yield

The two-stage pyrolysis coupled with nonthermal plasma processing was utilized to investigate the effect of plasma processing on the pyrolysis products derived from the pyrolysis of polystyrene, biomass waste, and a 1:1 mixture of the polystyrene and biomass. The product yield from pyrolysis alone and also in the presence of the nonthermal plasma at different input plasma powers of 50 and 70 W was investigated. Table 2 shows the gas, liquid (oil/water), and char yields.

**Table 2:** Product Yield from the Pyrolysis and Pyrolysis-Nonthermal Plasma Processing of Polystyrene, Biomass, and a Polystyrene/Biomass Mixture in Relation to Input Plasma Power

feedstock	input power (W)	char (wt %)	gas (wt %)	liquid (wt %)	oil (wt %)	water (wt %)
polystyrene	pyrolysis	1	0.39	98.61	98.61	0
	50	1	1.42	97.58	97.58	0
	70	1	3.93	95.07	95.07	0
biomass	pyrolysis	28.5	17.16	54.34	9.36	44.98
	50	28.5	18.26	53.24	8.76	44.48
	70	28.5	19.04	52.46	7.65	44.81
biomass–polystyrene	pyrolysis	13.75	8.04	78.21	51.12	27.09
	50	13.75	10.51	75.74	50.54	25.20
	70	13.75	11.44	74.81	50.12	24.69

The pyrolysis of polystyrene in the absence of plasma produced a high oil yield of 98.61 wt % linked to the high pyrolysis volatile content of plastics.^33^ Liu et al.^34^ similarly reported a high oil yield of 90.7 wt % from the pyrolysis of polystyrene at 650 °C in a fluidized-bed reactor, and Stančin et al.^33^ obtained 96.02 wt % oil yield for the pyrolysis of polystyrene at 600 °C using a thermogravimetric analyzer.

The introduction of the plasma for the pyrolysis-plasma processing of polystyrene produced an increase in gas yield from 0.39 wt % with no plasma (pyrolysis only) to 1.42 wt % at 50 W input plasma power and 3.93 wt %, at 70 W input plasma power. There was a consequent decrease in the oil yield. Aminu et al.,^35^ reported production of 10.7 wt % gas and 87.6 wt % oil from the pyrolysis/nonthermal plasma catalytic cracking of polystyrene at the input power of 80 W. Increasing the input power led to the formation of more gas, which confirms the cracking of the pyrolysis vapors into gases in the nonthermal plasma.^35^ Xiao et al.^36^ investigated the pyrolysis of polypropylene over zeolite ZSM-5 in a two-stage fixed-bed pyrolysis reactor with a DBD plasma reactor. They reported that, in the catalytic pyrolysis of polypropylene, for pyrolysis only, high amounts of oil (54 wt %), wax (26 wt %), and gas (20 wt %) were generated; however, at the input plasma power of 60 W, the gas yield increased, the wax yield declined dramatically, and the oil yield slightly decreased. They reported that the presence of the nonthermal plasma encouraged the conversion of heavy hydrocarbons to light hydrocarbons.^36^ Meng et al.^37^ used a DBD nonthermal-plasma rector for the degradation of tar produced in a fluidized-bed gasification reactor. As the applied voltage was raised, the specific energy density of the DBD reactor also increased. This is anticipated to elevate the energy level of the electrons within the reactor discharge space, thereby substantially enhancing the likelihood of high-energy electron collisions with tar molecules. This, in turn, results in a more effective removal of tar. Nguyen et al.^38^ worked on the decomposition of high-density polyethylene to hydrogen and light hydrocarbons using nonthermal plasma. They found that the total gas yield increased from 6 to 9 mmol g^–1^_Plastic_ as the plasma power was raised from 10 to 60 W. The rise in plasma power led to a consistent increase in the formation rate of gaseous products. This trend can be linked to the presence of more plasma-active species, consequently accelerating the reaction kinetics.

Pyrolysis of biomass generated 28.5% char, 17.16% gas, and 54.34% liquid (Table 2). Van Nguyen et al.^39^ produced 48.83% bio-oil, 31.29% char, and 19.88% gas during pyrolysis of pine sawdust. Chen et al.^19^ worked on pyrolysis of newspaper and reported formation of about 30% solid, 30% gas, and 40% liquid from pyrolysis of newspaper at 500 °C. The distribution of products from pyrolysis of biomass is different in literature reports because the amount of cellulose, hemicellulose, and lignin present in the biomass has a major influence on the distribution of biomass pyrolysis products. A higher proportion of lignin leads to increased char yield, whereas cellulose contributes to higher liquid and gas yields.^33^ In this work, the amount of cellulose would be high due to the high content of newspaper (42 wt %) and cardboard (42 wt %) in the biomass mixture used. Increasing the input plasma power from 50 to 70 W led to the formation of more gas and less liquid, as was the case for polystyrene. Blanquet et al.^5^ reported a 21% decrease in hydrocarbon tar content for a pyrolysis-nonthermal plasma process in comparison to a pyrolysis–catalysis process for the catalytic steam reforming of biomass. They also showed that compared to catalytic steam reforming without plasma plasma processing of the biomass, pyrolysis volatiles increased the overall gas production significantly. Wang et al.^40^ investigated biomass pyrolysis at a temperature of 550 °C coupled with postpyrolysis plasma reforming at 250 °C and reported that gas yield increased with rising input plasma power from 0 to 15 W.

Copyrolysis of biomass–polystyrene in the absence of plasma (pyrolysis) resulted in a product yield of 13.75% char, 8.04% gas, and 78.21% liquid. In comparison, when the nonthermal plasma was introduced at the input power of 50 W, there was an increase of 2.47% in gas yield, whereas a corresponding decrease was observed in liquid yield. Increasing the input plasma power to 70 W led to higher gas and less liquid production. The liquid phase was composed of two phases, water and oil, obtained from polystyrene and biomass. Xu et al.^41^ examined the effect of plasma and plasma-catalysis on tar reduction with the reforming temperature of 500 °C, discharge power of 15W, steam velocity of 6 mL/h/g_biomass_, and Ni-Fe/-Al_2_O_3_ as catalyst. The application of plasma discharge, when compared with only thermal heating at 500 °C, resulted in a moderate decrease of tar yield. This was attributed to the increasing influence of the nonthermal plasma characteristics in relation to the temperature, thereby affecting the efficiency of tar removal. The combined system featuring synergy between plasma and catalysis demonstrated the most effective results for eliminating tar. This success was attributed to the collaborative catalytic effects of a bimetallic catalyst and the nonthermal plasma, particularly through the processes of thermal cracking and reforming. Rutkowski et al.^42^ researched copyrolysis of cellulose and polystyrene at 500 °C and reported yields of 18.4% char, 22.8% gas, and 58.8% liquid when polystyrene and cellulose were mixed with equal mass. Kumar et al.^43^ studied copyrolysis of lignocellulosic biomass and polystyrene (1:1 ratio) at 510 °C and obtained an oil yield of 58 wt % and gas yield of 20 wt %. Stančin et al.^33^ performed copyrolysis of waste biomass sawdust (oak, poplar, and fir wood) and waste polystyrene at 600 °C using a thermogravimetric analyzer linked to a gas chromatograph coupled with off-line analysis of product oils. When the ratio of polystyrene and biomass was 1:1, around 75% liquid and 15 wt % gas were produced. Fan et al.^30^ explored the catalytic copyrolysis of cellulose and polyethylene using vacuum pyrolysis with a nonthermal plasma reactor system. They concluded that increasing the amount of plastic provided more hydrogen and carbon and also raised liquid yield and diminished gas yield.

Increasing the input plasma power will influence the reaction temperature in the plasma discharge zone.^41^ Xu et al.^41^ investigated the effect of plasma temperature on gaseous products from ambient temperature to 500 °C at a discharge power of 15W and steam velocity of 6 mL/h/g_biomass_ without a catalyst. The highest yields of all gaseous products, including H_2_, were achieved at the temperature of 200 °C. Nonthermal plasma without additional heating showed the highest hydrogen and total gas yields after 200 °C, which could be attributed to the self-heating effect caused by the plasma discharge. This can raise the temperature of the reactor from 100 to several hundred degrees depending on the energy input. Wang et al.^40^ studied biomass pyrolysis followed by nonthermal plasma reforming for hydrogen production. They examined different reforming temperatures in the nonthermal plasma reactor in the range of 250 to 550 °C at the input power of 15 W and found the maximum H_2_ yield at a temperature of 250 °C. Choosing lower temperatures is favored to boost the role of the nonthermal plasma in the reforming process. This preference arises because, at higher temperatures, lower mean electric fields are produced across the discharge gap, suggesting that the mean electron energy density was decreased.^40^ Consequently, these low-energy electrons are not likely to break the molecular bonds of the pyrolysis volatiles. Gao et al.,^44^ reviewed the DBD plasma-assisted catalytic dry reforming of methane. They reported that, in a DBD plasma reactor, elevating the input power corresponded to an increase in the conversion rates of CH_4_ and CO_2_. This phenomenon occurs because heightened input power results in an augmented electron density, accelerating collisions between reaction gas molecules and high-energy electrons. Consequently, this acceleration promotes the activation of reactants. The excited, dissociated, and ionized molecules of the reactants then initiate the dry reforming reaction of methane.^44^ Liu et al.^45^ studied the reforming of toluene in a nonthermal-plasma system. They concluded that conversion efficiency of toluene was elevated with increasing input plasma power from 39 to 90 W. They attributed this to the rise in the number of microdischarges achieved through increased discharge power. This enhancement promotes the generation of additional reaction channels and reactive species during toluene reforming, consequently leading to an elevated level of toluene conversion.^45^ Taghvaei et al.^46^ observed that an increase in voltage resulted in an enhancement in discharge power and guaiacol conversion during hydrodeoxygenation of guaiacol in a DBD nonthermal plasma reactor. Higher applied voltages lead to the creation of a more powerful electric field and stronger microdischarges, resulting in increased energy and electron density in the discharge zone. Consequently, the likelihood of electron impact dissociation reactions involving processes such as ionization, excitation, and dissociation of gas molecules is heightened. As a result, there is an enhanced probability of breaking guaiacol chemical bonds due to the increased number and effectiveness of collisions with reactive species.

### 3.2 Gas Composition

**Figure 3:**
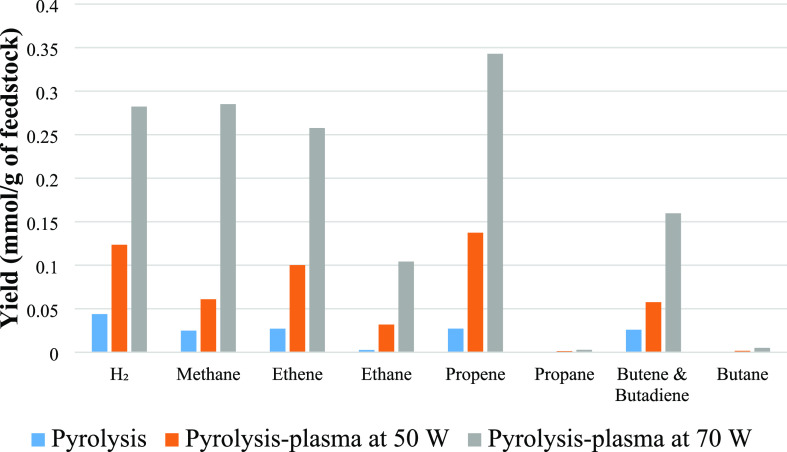
Gas composition from the pyrolysis and pyrolysis-nonthermal plasma processing of polystyrene in relation to input plasma power.

The gas composition was investigated for the pyrolysis of polystyrene, biomass, and their 1:1 mixture under pyrolysis conditions and also in the presence of the nonthermal plasma at the input powers of 50 and 70 W. Figure 3 shows the gas yields from the pyrolysis (no plasma) and the pyrolysis-plasma processing of polystyrene at the input powers of 50 and 70 W. The yields of all gas products were increased as input plasma power was increased. Hydrogen yield produced from processing polystyrene was increased from 0.04 mmol g^–1^ for pyrolysis alone to 0.12 mmol g^–1^ for pyrolysis-plasma at 50 W and to 0.28 mmol g^–1^ at the input power of 70 W. Methane also increased from 0.02 mmol g^–1^ for pyrolysis to 0.28 mmol g^–1^ at 70 W. Wang et al.^40^ studied a two-stage pyrolysis-DBD plasma process for H_2_ production from cellulose at a pyrolysis temperature of 550 °C and a reforming temperature of 250 °C. They reported an increase of all gas yields including CO, CO_2_, CH_4_, C_2_–C_3_, and H_2_ with an increase of plasma input power from 0 to 15 W. Increasing input power to 70 W led to more gas production. Nguyen et al.^38^ confirmed that introducing a nonthermal-plasma stage to the thermal stage outperformed the thermal case, which can be attributed to the presence of vibrationally excited species. Increasing the input power raised the production of hydrogen and methane, which were the predominant components at higher input powers (40–60 W). Under high plasma power conditions, hydrogen emerged as the predominant product. This was attributed to the endothermic cracking of long-chain C–H fragments, a process promoted by the elevated temperatures observed at high plasma power levels. Saleem et al.^47^ examined the reactions of toluene in a DBD nonthermal plasma reactor with H_2_ as the carrier gas. With the rise in plasma power, the efficiency of toluene decomposition increased, and also the overall selectivity toward lower hydrocarbons and the selectivity of C_1_–C_5_ hydrocarbons increased with higher power levels, indicating the breakdown of the aromatic ring at elevated plasma power.

**Figure 4:**
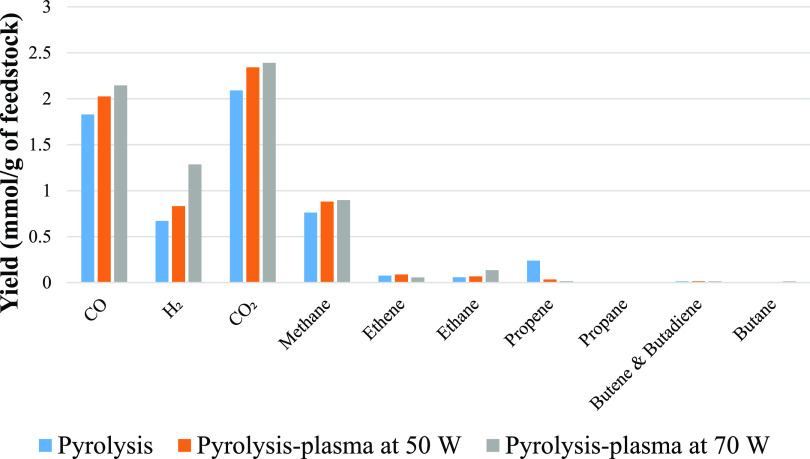
Gas composition from the pyrolysis and pyrolysis-nonthermal plasma processing of biomass in relation to input plasma power.

The gas yields from pyrolysis (no plasma) and also coupled pyrolysis and nonthermal plasma processing of biomass at 50 and 70 W are shown in Figure 4. The thermal degradation of biomass led to the formation of CO and CO_2_ derived from the oxygenated components of the biomass and constituted the main gases produced in the pyrolysis and pyrolysis-plasma process. Also produced were H_2_ and CH_4_ along with lower concentrations of C_2_–C_4_ hydrocarbons. Increasing the input plasma power resulted in higher gas yields. The increased production of CO and CO_2_ at higher input powers suggests that deoxygenation reactions of the biomass derived pyrolysis volatiles occur in the form of decarboxylation and decarbonylation of the oxygenated hydrocarbons during the nonthermal plasma process. Blanquet et al.^48^ researched H_2_ production from waste biomass via a pyrolysis-nonthermal plasma-catalytic reforming processing. They also reported that the yields of hydrogen, methane, carbon monoxide, and carbon dioxide increased as input plasma power was increased from 40 to 80 W.

The interaction of the pyrolysis gases with the nonthermal plasma produced an increase in gas yield that may be attributed to the enhanced cracking of the higher-molecular-weight pyrolysis hydrocarbon volatiles to lower-molecular-weight species by the high-energy electrons and other energetic species in the plasma environment. For example, Fridman^49^ reported that the electrons generated in a nonthermal plasma have higher energy than the dissociation energies of C–H and C–C chemical bonds.

**Figure 5:**
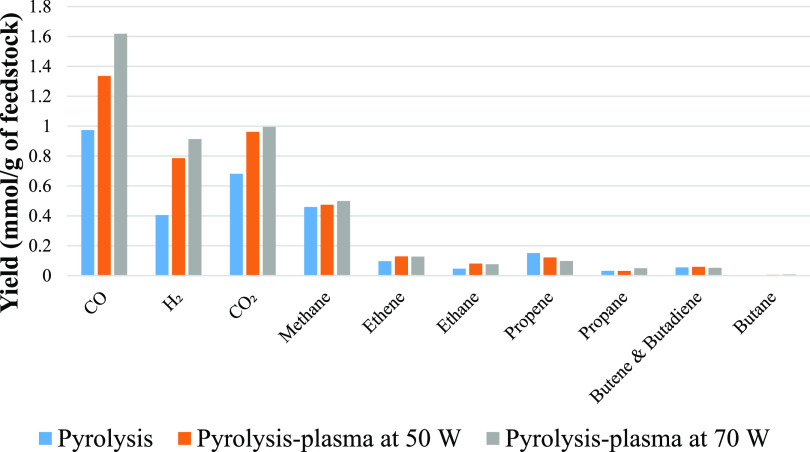
Gas composition from the pyrolysis and pyrolysis-nonthermal plasma processing of a polystyrene/biomass mixture in relation to input plasma power.

The gas yields from the copyrolysis and also copyrolysis coupled with nonthermal plasma processing of the 1:1 mixture of polystyrene and biomass at input plasma powers of 50 and 70 W are shown in Figure 5. CO and CO_2_ are the dominant gases for pyrolysis only and for the pyrolysis-plasma system, indicating that decarboxylation and decarbonylation of the biomass pyrolysis volatiles occurred, and their amount was enhanced at higher input plasma power. Hydrogen, methane, ethane, ethane, propane, and butane were generated in the process of pyrolysis and were further increased in yield as the nonthermal plasma was introduced. Titov et al.^50^ explored nonthermal plasma pyrolysis of fuel oil at low temperatures and concluded that with a rise in voltage from 300 to 700 V, the amount of hydrogen, methane, ethane, ethane, propane, and butane increased. Xu et al.^41^ investigated reforming of pyrolysis volatiles derived from biomass using a combined pyrolysis and plasma-catalysis technology for H_2_ production. They carried out experiments at different discharge powers in the nonthermal plasma reactor in the absence of a catalyst and steam. The introduction of plasma was found to significantly increase the yields of all gaseous products, particularly for the production of H_2_ and CO, when compared to the thermal-only condition. The observed outcomes were primarily attributed to the higher abundance of electrons generated through intensified microdischarges in the catalytic nonthermal plasma process. This increase in electrons offers additional reaction channels and reactive species, thereby further facilitating more reaction processes.^41^ Meng et al.^37^ investigated the gas and tar composition produced from a fluidized-bed gasification of coal processed through a DBD plasma reactor. They found that increasing the specific energy density led to the formation of more alkanes and less aromatics in the gas phase. Within the plasma discharge, aromatic hydrocarbons and other cyclic substances undergo conversion into aliphatic hydrocarbons, as they are subjected to the impact of high-energy electrons.

### 3.3 Oil Composition

Table 3 shows the oil components present in the product oil from the pyrolysis-plasma processing of polystyrene for pyrolysis only and for the coupled pyrolysis-plasma processing at input plasma powers of 50 and 70 W. The compounds in Table 3 were identified using GC–MS. For pyrolysis only and also for the pyrolysis coupled nonthermal plasma processing at the input power of 50 W, the main components were styrene, 2-phenyl-1,2,3,4-tetrahydronaphthalene, 2,4-dimethyl-1-heptene, and 6-tridecene, respectively. Increasing the input plasma power to 70 W resulted in a large increase in the yields of ethylbenzene, toluene, and methylstyrene, whereas styrene yield was almost constant. It is also noticeable that the amount of 2-phenyl-1,2,3,4-tetrahydronaphthalene was halved as input plasma power was increased to 70 W. This can be attributed to the fact that increasing the applied voltage causes a rise in the amounts and energy levels of electrons and ions, which ultimately results in the breakdown of stronger chemical bonds.^51^ Hosseinzadeh et al.^51^ focused on upgrading a lignin bio-oil model compound in the form of 4-methylanisole using a DBD plasma reactor. They reported selectivity for 4-methylphenol, 2,4-dimethylphenol, and 1-ethoxy-4-methylbenzene that tended to decrease as the voltage was raised; however, the trend for other lighter compounds tended toward an increase.

**Table 3:** Yield of Polystyrene-Derived Oil Compounds (mg/g of Feedstock) from Pyrolysis-Plasma at 0, 50, and 70 W Plasma Power

**no.**	**RT (min)**	**peak name**	**M.W.**	**pyrolysis**	**pyrolysis-plasma at** 50 W	**pyrolysis-plasma at** 70 W
1	2.671	benzene	78	3.14	1.43	7.15
2	4.661	toluene	92	38.63	33.99	78.27
3	7.529	2,4-dimethyl-1-heptene	126	55.98	54.40	40.37
4	8.433	ethylbenzene	106	23.43	27.94	84.72
5	8.91	*p*-xylene	106	0.00	0.30	0.70
6	10.175	styrene	104	516.87	540.88	509.38
7	15.831	methylstyrene	118	21.27	21.44	37.37
8	18.935	benzene, 2-propenyl-	118	0.00	0.00	3.81
9	21.011	benzene, (1-methylenepropyl)-	132	0.00	0.00	4.42
10	25.584	unknown		5.79	3.88	0.00
11	26.954	naphthalene	128	0.22	0.15	0.60
12	31.116	3-tridecene	182	38.88	36.36	0.00
13	31.295	unknown		20.23	14.24	7.59
14	31.621	6-tridecene	182	48.72	44.51	0.00
15	33.051	benzene, (1-methylenepentyl)-	160	5.57	5.04	0.00
16	35.807	unknown		7.59	5.19	2.43
17	37.009	bibenzyl	182	4.53	4.15	5.53
18	37.747	benzene, 1,1′-(1-methyl-1,2-ethanediyl)bis	196	3.37	3.43	4.96
19	38.546	benzene, 1,1′-(1,2-dimethyl-1,2-ethanediyl)bis-	210	0.00	1.50	1.84
20	39.197	benzene, (1-methylhexyl)	176	0.00	1.85	2.48
21	39.708	unknown		13.11	9.17	5.50
22	40.159	benzene, 1,1′-(1,3-propanediyl)bis-	196	10.70	10.77	8.73
23	40.547	benzene, 1,1′-cyclopropyldenebis-	194	0.00	0.00	2.30
24	40.785	benzene, 1,1′-(1-methyl-1,3-propanediyl)bis	210	0.00	0.00	2.52
25	41.029	benzene, 1,1′-(1-butenylidene)bis-	208	0.00	0.00	2.58
26	41.46	1,2-diphenylethylene	180	0.00	0.00	2.52
27	41.636	2-phenyl-1,2,3,4-tetrahydronaphthalene	208	91.42	86.72	54.43
28	41.768	benzene, 1,1′-(1,2-ethanediyl)bis[4-methyl-	210	5.62	5.69	6.29
29	41.857	1-octadecene	252	0.00	0.00	12.61
30	42.008	1,2-diphenylcyclopropane	194	3.65	3.49	5.52
31	42.47	benzene, 1,1′-(1,4-butanediyl)bis-	210	0.00	2.36	2.97
32	42.97	phenanthrene	178	0.00	0.00	0.43
33	43.047	unknown		19.57	14.10	11.98
34	43.16	naphthalene, 1,2-dihydro-4-phenyl-	206	0.00	0.00	3.61
35	43.378	benzene, 1,1′-(3-methyl-1-propene-1,3-diyl)bis-	208	3.90	3.01	4.72
36	43.857	benzene, 1,1′-(1-butene-1,4-diyl)bis-, (z)-	208	0.00	3.14	0.00
37	43.957	benzene, 1,1′-(1-ethenyl-1,3-propanediyl)bis-	222	0.00	3.46	0.00
38	45.229	1-nonadecene	266	6.01	5.18	6.24
39	46.019	unknown		9.82	6.41	7.21
40	47.1	1-(4-methylphenyl)-4-phenylbuta-1,3-diene	220	0.00	2.88	5.39
41	48.736	unknown		5.13	3.92	5.32
42	51.206	unknown		7.28	0.00	0.00
43	54.966	unknown		15.62	14.85	8.19

**Figure 6:**
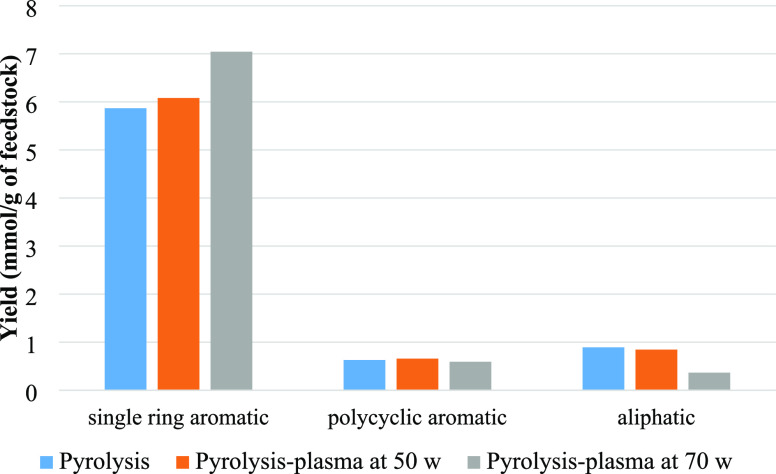
Yield (mmol/g of feedstock) of aliphatic, polycyclic, and monocyclic aromatic groups from pyrolysis alone and pyrolysis-plasma processing of polystyrene at 50 and 70 W.

Figure 6 shows the yield of different chemical groups in the oil obtained from the pyrolysis alone and pyrolysis-plasma processing of polystyrene at the input plasma powers of 50 and 70 W. It can be seen that when the nonthermal plasma was introduced at the input plasma power of 50 W, the yield of monocyclic aromatic compounds slightly increased in concentration, and raising the input plasma power to 70 W led to more production of single-ring aromatic compounds. The content of polycyclic aromatic hydrocarbons (PAH) was similar for pyrolysis and the pyrolysis-plasma processing at 50 W but then showed a decline from 0.66 to 0.59 mmol/g_feedstock_ when input plasma power was increased to 70 W. The large yields of aromatic compounds are caused by the thermal breakdown of polystyrene, which results in radical chain end scission of the polymer chain subsequently followed by depolymerization or intramolecular hydrogen abstraction. Depolymerization is the main way to generate monomer molecules; however, oligomers like dimers and trimers are formed via a backbiting process followed by β-scission.^52^ Song et al.^53^ researched pyrolysis-DBD plasma-catalysis of polyethylene for the production of light aromatic compounds and claimed that the selectivity of monoaromatic compounds was improved as the plasma power was increased. Raising power increased the production of short-chain hydrocarbons and slowed down the monocyclic aromatic hydrocarbon condensation reaction during the polyethylene scission reaction.^53^ Fan et al.^31^ working on the pyrolysis of camphor wood sawdust catalyzed by HZSM-5 suggested that the use of nonthermal plasma technology enhanced the production of monocyclic aromatic hydrocarbons and decreased the amount of polycyclic aromatic hydrocarbons in the oil. Xu et al.^41^ reported on the pyrolysis of biomass followed by different reforming modes of postprocessing, plasma, catalyst, and plasma-catalysis and classified the compounds in the product tar to the different modes in terms of their carbon number as ≤C_10_, C_11_–C_20_, C_21_–C_30_, and >C_30_. The tar produced solely through the postprocessing heating mode primarily consisted of biomass tar formed during the pyrolysis stage. This tar comprised aromatic hydrocarbons, long-chain hydrocarbons, and oxygenated hydrocarbons, resulting from inadequate and efficient thermal decomposition at temperatures of 500 °C. Introducing the nonthermal plasma for the postprocessing of the biomass pyrolysis volatiles doubled the percentage of light compounds (≤C_10_) and decreased the higher-range hydrocarbon compounds in comparison to heating only. In the integrated pyrolysis and plasma-catalysis system, they proposed that the reactive species present in the plasma zone enhance the breaking of chemical bonds within the tar compounds, thereby generating gases and light hydrocarbons through collision and recombination.

Table 4 shows the yields of oil components produced from the pyrolysis of biomass (no plasma) and the pyrolysis-plasma processing of biomass at 50 and 70 W input plasma power. It should be noted that the total number of peaks identified and reported in Table 4 represents 71.1, 77.3, and 74.6% of all the total compounds identified in Table 4 for pyrolysis, pyrolysis-plasma at 50 W, and pyrolysis-plasma at 70 W, respectively. That is, for each scenario, between 28.9 and 22.7% of the peaks were too low in concentration to be identified and reported. Methylglyoxal was the main compound present in the product bio-oil at all input plasma powers. Chen et al.^54^ also reported the formation of methylglyoxal for the fast pyrolysis of waste newspaper. Blanquet et al.^5^ reported the formation of alkylated phenols or aromatic compounds, including benzene, phenol, *o*-cresol, *p*/*m*-cresol, guiaicol, 2,4-dimethylphenol, 4-ethylphenol, 4-isopropylphenol, and 2- methoxy-4-propylphenol during pyrolysis–catalysis, pyrolysis nonthermal plasma, and pyrolysis-nonthermal plasma-catalysis for the steam reforming process of biomass. They concluded that the concentration of the individual aromatic and oxygenated aromatic hydrocarbons was decreased during the plasma and plasma-catalytic processes. The largest reduction in the hydrocarbon concentration was achieved by the plasma-catalytic process.

**Table 4:** Yield of Biomass-Derived Oil Compounds (mg/g of Feedstock) from Pyrolysis Alone and Pyrolysis-Plasma Processing at 50 and 70 W

**no.**	**RT (min)**	**peak name**	**M.W.**	**pyrolysis**	**pyrolysis-plasma at** 50 W	**pyrolysis-plasma at** 70 W
1	2.673	methylglyoxal	72	41.66	44.88	39.07
2	3.124	propanal, 2,3-dihydroxy-	90	3.08	4.16	4.10
3	3.285	propanoic acid, 2-hydroxy-	90	5.09	6.86	7.02
4	3.684	acetic acid, 1-methylethyl ester	102	5.02	6.88	5.91
5	4.629	3-pentanone	86	3.13	3.02	2.45
6	4.760	acetic acid, 1-methylester	74	6.61	5.51	4.18
7	5.368	acetohydroxamic acid	75	7.05	7.97	7.44
8	5.634	cyclopentanone	84	5.06	0.89	0.75
9	7.057	furfural	96	2.25	1.21	0.76
10	8.146	2-furanmethanol	98	0.34	0.00	0.00
11	8.238	butanal, 2-ethyl-	100	0.43	0.27	0.21
12	8.895	acetic anhydride	102	0.33	0.42	0.39
13	10.746	furan, 2,4-dimethyl-	96	0.14	0.20	0.15
14	10.833	unknown		0.52	0.00	0.00
15	10.945	2-furanone	84	0.40	0.24	0.21
16	11.079	furan, 2-ethyl-5-methyl	110	0.28	0.30	0.27
17	11.488	unknown		0.34	0.11	0.08
18	11.782	octane, 2,3-dimethyl-	142	1.09	0.00	0.00
19	14.382	2-furancarboxaldehyde, 5-methyl-	110	0.52	0.45	0.30
20	14.704	propanoic acid, ethenyl ester	100	0.29	0.25	0.21
21	14.915	2-butanone, 1-(acetyloxy)-	130	0.18	0.19	0.16
22	15.915	phenol	94	0.85	1.31	1.22
23	16.598	1,2-cyclopenten-1-one, 2-hydroxy-3-methyl-	112	2.34	0.00	0.00
24	18.693	1,2-cyclopentanedione, 3-methyl-	112	2.43	0.95	0.60
25	21.426	acetophenone	120	0.04	0.08	0.07
26	21.565	phenol, 2-methoxy-	124	0.16	0.00	0.00
27	22.581	mequinol	124	0.21	0.13	0.10
28	26.877	phenol, 2-methoxy-4-methyl-	138	0.96	0.00	0.00
29	27.182	phenol, 2-methoxy-5-methyl-	138	2.56	0.99	0.64
30	30.117	phenol, 4-ethyl-2-methoxy-	152	0.18	0.28	0.17
31	33.665	vanillin	152	0.04	0.02	0.03

**Figure 7:**
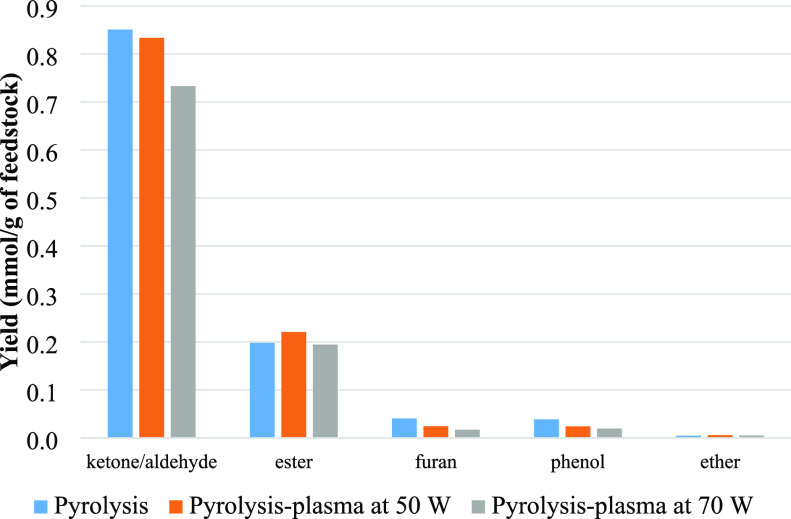
Yield (mmol/g of feedstock) of oxygenated groups from pyrolysis alone and pyrolysis-plasma processing of biomass at 50 and 70 W.

Figure 7 shows the yields of oxygenated groups, including ketone/aldehyde, ester, furan, and phenol groups, identified in the product bio-oil in terms of mmol g^–1^ of biomass feedstock. Cellulose pyrolysis provides monosaccharides, alcohols, aldehydes, ketones, ethers, esters, and furans.^55^ These groups were generated during pyrolysis-plasma processing of biomass for pyrolysis only and pyrolysis-plasma processing at 50 and 70 W input plasma power. Ketones, furans, and phenols declined in yield as the input plasma power was raised, which confirm that the deoxygenation process occurred when applying the plasma. These findings are consistent with research carried out by Taghvaei et al.,^46^ who found that the higher the applied voltage was, the greater was the degree of deoxygenation in relation to guaiacol through DBD plasma upgrading. In the study conducted by Taghvaei et al.,^46^ hydrodeoxygenation reactions were carried out using a quartz glass DBD tubular reactor with argon as the carrier gas for upgrading guaiacol, a representative model compound of lignin pyrolysis oil. Hydrogen was generated *in situ* by applying a discharge plasma power of 100 W, leading to the decomposition of methyl and methoxyl radicals derived from the lignin pyrolytic oil. Mono-oxygenated compounds—specifically, phenol, methylphenols (2-methylphenol and 4-methylphenol), and dimethylphenols (2,4–2,6 and 3,4-dimethylphenol)—along with benzene, toluene, and xylene were identified as products of the decomposition. Additionally, trace amounts of anisole, catechol, methylanisoles, cyclohexanol, and trimethylphenols were detected.

**Figure 8:**
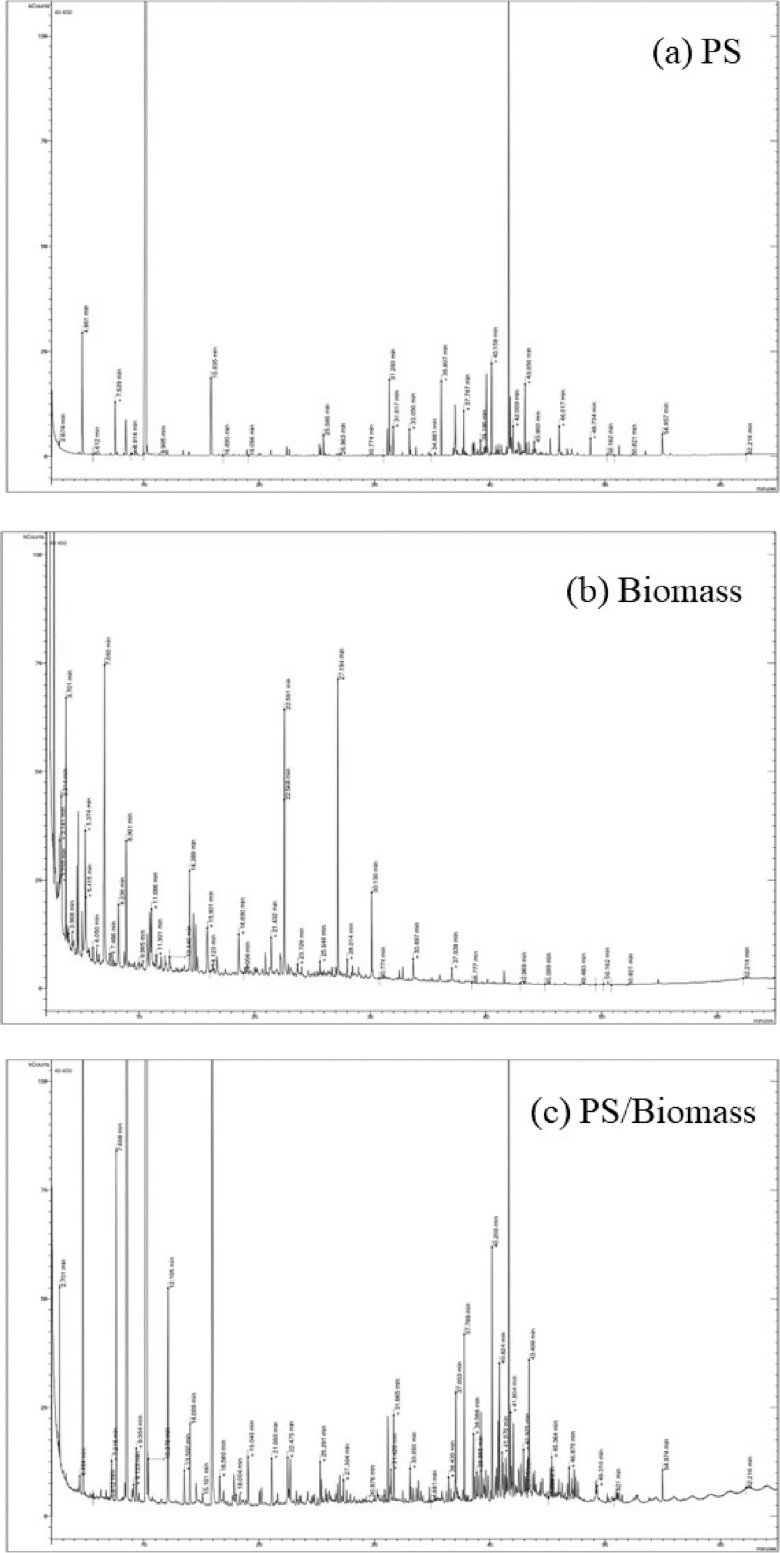
Total ion chromatograms (TICs) of the product oils from the pyrolysis coupled with nonthermal plasma processing (a) polystyrene, (b) biomass, and (c) 1:1 mixture of polystyrene and biomass.

Figure 8 shows the total ion chromatograms (TICs) obtained from the analysis of the product oils by GC–MS in relation to the pyrolysis with nonthermal plasma processing of polystyrene (Figure 8a) and biomass (Figure 8b) and the copyrolysis-plasma processing of a 1:1 mixture of polystyrene and biomass (Figure 8c). It can be observed by comparison of the three TICs that, visually, there appears to be an interaction of the polystyrene and biomass when the two feedstocks are processed together by copyrolysis followed by nonthermal plasma reaction. Several TIC peaks are either markedly reduced or increased in the TIC of the 1:1 mixture compared to what might be expected by mere mixing of the product oils from polystyrene and biomass. Table 5 shows the composition of the product oils produced from the copyrolysis only and copyrolysis-plasma processing of the polystyrene and biomass 1:1 mixture at 50 and 70 W input plasma power. The compounds reported in Table 5 represent a high proportion of all the compounds present in the oil at 97.5, 93.4, and 81% of the total produced liquid for pyrolysis, pyrolysis-plasma at 50 W, and pyrolysis-plasma at 70 W, respectively. The remaining percentages represent numerous compounds of low concentration. Most of the compounds produced in the copyrolysis of polystyrene and biomass were those produced from the pyrolysis of polystyrene. This suggests that the plastic is dominant for the yield of oil components.^33^ The main oil compounds from the copyrolysis (no plasma) of the polystyrene and biomass are styrene, methylglyoxal, ethylbenzene, and 2-phenyl-1,2,3,4-tetrahydronaphthalene. Meanwhile, ethylbenzene, styrene, 2,4-dimethyl-1-heptene, 6-tridecene, and alpha-methylstyrene were the dominant compounds for the copyrolysis-plasma processing at 50 and 70 W plasma input power.

**Table 5:** Yield of Biomass-Polystyrene-Derived Oil Compounds (mg/g of Feedstock) from Pyrolysis Alone and Pyrolysis-Plasma Processing at 50 and 70 W Input Plasma Power

**no.**	**RT (min)**	**peak name**	**M.W.**	**pyrolysis**	**pyrolysis-plasma at** 50 W	**pyrolysis-plasma at** 70 W
1	2.689	benzene	78	0.00	7.36	5.68
2	2.691	methylglyoxal	72	108.57	0.00	0.00
3	4.701	toluene	92	0.01	0.01	0.01
4	7.187	furan, 2,5-dimethyl	96	1.71	0.00	0.00
5	7.214	cyclopentane, 1,1,3,4-tetramethyl-, trans	126	0.00	0.00	0.00
6	7.588	2,4-dimethyl-1-heptene	126	20.78	24.03	21.97
7	8.496	ethylbenzene	106	62.94	197.49	185.23
8	9.354	phenylethyne	102	0.00	1.96	2.90
9	10.188	styrene	104	197.87	169.99	167.71
10	10.375	2,3,3-trimethyl-1-hexene	126	0.00	2.61	6.90
11	12.083	benzene, (1-methylethyl)-	120	1.24	3.90	5.30
12	13.488	benzene, 1-ethenyl-2-methyl-	118	0.71	0.69	1.41
13	14.010	benzene, propyl-	120	0.00	1.60	2.65
14	14.539	phenylglyoxal	134	0.00	0.91	1.55
15	15.909	alpha-methylstyrene	118	12.08	11.66	13.53
16	16.579	*cis*-beta-methylstyrene	118	0.00	0.74	1.14
17	17.820	benzene, (1-methylpropyl)-	134	0.00	0.65	1.26
18	19.028	benzene, 2-propenyl-	118	1.37	1.16	1.70
19	20.023	benzene, 1-propynyl-	118	0.70	0.46	0.64
20	21.087	benzene, (1-methylenepropyl)-	132	1.36	1.27	1.80
21	22.461	3-undecene	154	6.74	6.29	6.86
22	22.699	4-undecene	154	6.31	6.48	7.82
23	25.273	benzene, (3-methyl-3-butenyl)-	146	0.90	0.64	0.89
24	25.398	benzene, (1-ethyl-2-propenyl)-	146	0.72	0.00	0.70
25	27.295	phenol, 2-methoxy-5-methyl-	138	0.00	0.85	1.64
26	31.147	3-tridecene	182	11.13	9.53	11.02
27	31.401	unknown		1.62	0.00	1.16
28	31.648	6-tridecene	182	16.11	19.05	12.79
29	33.081	benzene, (1-methylenepentyl)-	160	0.90	0.58	0.76
30	35.842	unknown		0.31	0.11	0.00
31	36.186	1,1′-biphenyl, 3-methyl	168	0.41	0.00	0.00
32	37.038	bibenzyl	182	1.12	0.99	1.00
33	37.776	benzene, 1,1′-(1-methyl-1,2-ethanediyl)bis	196	0.80	1.11	1.20
34	38.586	benzene, 1,1′-(1,2-dimethyl-1,2-ethanediyl)bis-	210	0.00	0.55	0.77
35	39.037	alpha-methylstilbene	194	0.00	0.49	0.00
36	39.238	benzene, (1-methylhexyl)	196	0.34	0.91	1.33
37	39.655	benzene, 1,1′-(2-butene-1,4-diyl)bis-	208	0.00	0.30	0.66
38	39.842	unknown		0.00	0.46	0.64
39	39.829	hexane, 3,4-diphenyl-	238	0.00	0.46	0.64
40	40.186	benzene, 1,1′-(1,3-propanediyl)bis-	196	2.75	2.41	2.64
41	40.580	benzene, 1,1′-cyclopropyldenebis-	194	0.00	0.39	0.63
42	40.728	benzene, 1,1′-(2-methyl-1-propeneylidene)bis-	208	0.37	0.74	0.83
43	40.824	benzene, 1,1′-(1-methyl-1,3-propanediyl)bis-	210	0.42	1.54	1.57
44	41.070	benzene, 1,1′-(1-butenylidene)bis-	208	0.00	0.54	0.97
45	41.148	1-heptadecene	238	0.00	2.06	0.00
46	41.394	benzene, 1,1′-(1,1,2,2-tetramethyl-1,2-ethanediyl)bis	238	0.00	0.44	0.75
47	41.501	1,2-diphenylethylene	180	0.58	0.39	0.64
48	41.647	2-phenyl-1,2,3,4-tetrahydronaphthalene	208	22.32	7.61	6.11
49	41.804	benzene, 1,1′-(1,2-ethanediyl)bis[4-methyl-	196	0.00	0.70	1.00
50	42.032	1,2-diphenylcyclopropane	194	1.29	0.63	1.19
51	42.500	benzene, 1,1′-(1,4-butanediyl)bis-	210	0.55	0.47	0.89
52	42.914	benzene, 1,1′-(1,2-dimethyl-1,2-ethanediyl)bis-	208	0.66	0.60	0.97
53	43.050	1-(4-methylphenyl)-4-phenylbuta-1,3-diene	220	1.25	0.00	0.78
54	43.185	naphthalene, 1,2-dihydro-4-phenyl-	206	0.77	0.52	0.00
55	43.398	benzene, 1,1′-(3-methyl-1-propene-1,3-diyl)bis-	208	1.94	2.99	2.86
56	43.744	naphthalene, 1,2-dihydro-3-phenyl-	206	0.69	0.80	0.78
57	43.875	benzene, 1,1′-(1-butene-1,4-diyl)bis-	208	1.03	0.60	0.84
58	44.553	naphthalene, 1-phenyl	204	0.64	0.44	1.34
59	45.248	1-nonadecene	266	2.56	0.83	0.73
60	45.364	2-nonadecene	266	0.00	2.08	0.00
61	45.957	1-eicosene	280	0.83	0.47	0.57
62	46.725	unknown		1.41	0.39	0.46
63	46.865	2-phenylnaphthalene	204	0.76	0.61	0.76
64	47.106	unknown		1.59	0.46	0.73
65	47.636	eicosane	282	0.82	0.43	0.49
66	54.957	unknown		9.48	0.97	1.41
67	55.235	unknown		1.70	0.00	0.00

In this work, no nitrogen-containing hydrocarbon species were detected in the product oil. They may have been present in the oil at very low concentrations, formed from nitrogen in the feedstock (Table 1) or formed from nitrogen carrier gas. Liu et al.^45^ have reported the formation of nitrogen-containing aromatic compounds such as 2-propen-1-amine, benzonitrile, and 3-methylbenzonitrile from the reforming of toluene in a DBD nonthermal-plasma reactor without steam and in the presence of 3 vol % O_2_/97 vol % N_2_ carrier gas. However, others have suggested that nitrogen-containing hydrocarbons may not form from the nitrogen carrier gas. Wang et al.^40^ reported on cellulose pyrolysis and DBD plasma-assisted reforming for the production of H_2_. The existence of steam in the plasma process has the potential to generate H and OH radicals by dissociating water through the electrons produced by the plasma and excited nitrogen species, such as N_2_* from nitrogen. However, they suggested that the N_2_* reverts to N_2_ on reaction.

**Figure 9:**
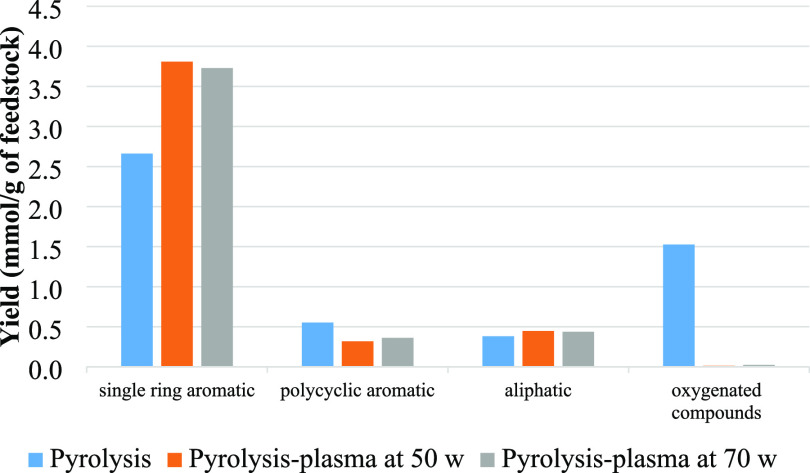
Yield (mmol/g of feedstock) of monoaromatic, polycyclic aromatic, aliphatic, and oxygenated groups from pyrolysis alone and pyrolysis-plasma processing of biomass and polystyrene at 50 and 70 W input plasma power.

Figure 9 classifies the single-ring aromatic, polycyclic aromatic, aliphatic, and oxygenated groups based on mmol g^–1^ of feedstock produced in copyrolysis and the coupled copyrolysis-plasma processing of polystyrene and biomass at 50 and 70 W input plasma power. The monoaromatic compounds have the highest yields for copyrolysis and also with the copyrolysis-plasma processing conditions. The addition of the nonthermal plasma processing at 50 W input plasma power led to higher monoaromatic production. Özsin et al.^52^ investigated pyrolysis of polystyrene and lignocellulosic biomass and reported that the liquid products contained a significant amount of benzene and its derivatives. They concluded that almost all of the aromatic compounds were produced by free radicals resulting from the thermal degradation of polystyrene. The yield of polycyclic aromatic compounds decreased when the nonthermal plasma was introduced at 50 W input plasma power. Applying the nonthermal plasma to the process resulted in the oxygenated compounds present in the product oil to become reduced to almost zero at 50 and 70 W input plasma power. Moreover, some hydrocarbons, including aromatic and aliphatic compounds, were produced with the introduction of the polystyrene to the biomass. These results are in accordance with findings reported by Fan et al.^30^ They reported that introducing polyethylene to biomass with an equal mass ratio decreased the concentration of oxygenated compounds in the product oil from ∼65 to 7% and increased the content of aromatic compounds. This process aided deoxygenation by transferring hydrogen radicals from the polyethylene-derived hydrocarbons to biomass-derived oxygenates. The presence of hydrogen-containing radicals derived from the pyrolysis of polyethylene was advantageous for removing oxygen from biomass-derived oxygenated compounds. This deoxygenation process was likely to produce highly reactive oxygen radicals when exposed to nonthermal plasma conditions. These oxygen radicals, in turn, facilitated the breaking down and decomposition of long-chain olefins or olefinic radicals.^30^ Liu et al.^56^ used a lignin-derived monomer and nonthermal plasma to investigate the upgrading of bio-oil and presented a mechanism for product selection via the correlation between product distribution and mean electron energy. They concluded that the reaction was either directed toward aromatic ring hydrogenation or hydrodeoxygenation, depending on the gas temperature. Increased voltage enhanced the mean electron energy, which aided in the elimination of oxygenated functional groups from the guaiacol. The main products were shifted from catechol to cresol and phenol and subsequently to benzene, toluene, and xylene.

### 3.4 Synergistic Effect

**Figure 10:**
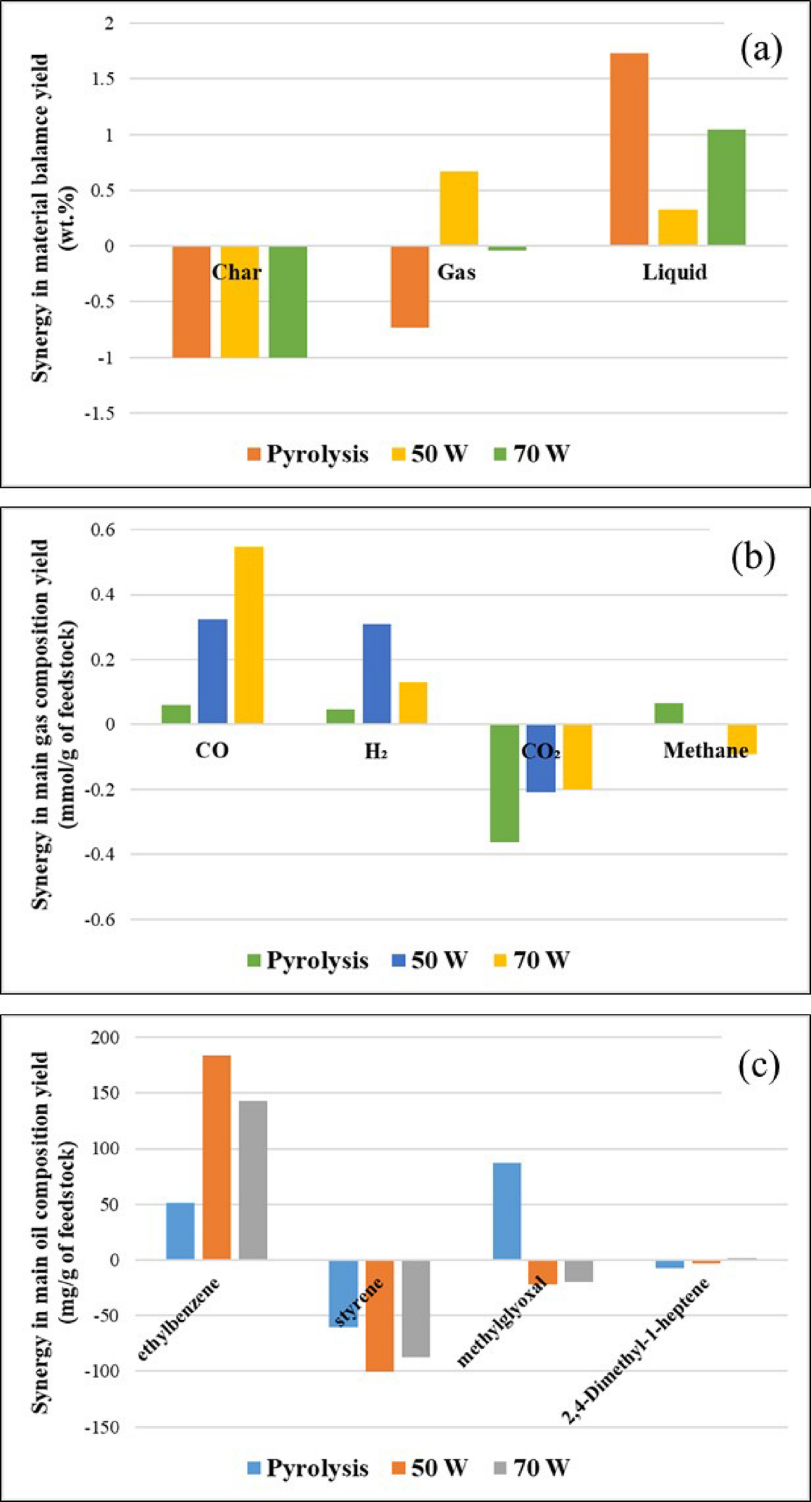
Synergistic effect on product yield for pyrolysis alone and the pyrolysis with nonthermal plasma processing of biomass and polystyrene: (a) product yield, (b) gas composition, and (c) oil composition yield of main components (mg/g of feedstock).

Figure 10 shows the synergistic effect (based on eq 7) in relation to the product yields (Figure 10a), gas yield (Figure 10b), and the yield of the main oil compounds (Figure 10c) for the pyrolysis alone and for the coupled pyrolysis-nonthermal plasma processing of biomass and polystyrene at input plasma powers of 50 and 70 W. These quantities were obtained from the difference between experimental and theoretical values. Figure 10a indicates that there were a small positive synergy effect on the liquid yield and a small negative effect on the char yield for pyrolysis and the pyrolysis-plasma processing. These results show that mixing biomass and polystyrene encouraged liquid production and reduced char production. These findings can be explained by the fact that polystyrene pyrolysis produces free radicals and donates hydrogen, both of which initiate a cross-reaction between biomass and polystyrene during copyrolysis.^57^ Experimental gas yield was lower than its theoretical additive value during the pyrolysis only process. This could be due to reactions of noncondensable fragments produced through secondary radical reactions during copyrolysis.^52^ Muneer et al.^57^ reported on the copyrolysis of biomass and polystyrene in a fixed-bed reactor and showed a synergistically produced increase in oil yield above that predicted by calculation of the individual feedstock pyrolysis, whereas there was a negative synergy on gas and char yields.

Figure 10b illustrates the synergistic effect on gas yields (mmol g^–1^ of feedstock) for the main gases produced during the pyrolysis only process and the coupled pyrolysis with nonthermal plasma processing of biomass and polystyrene at 50 and 70 W input plasma power. There was an increasing trend for the synergistic effect of the carbon monoxide yield with increasing input power. The synergistic effect on hydrogen yield was also positive, indicating that there is an interaction between biomass and polystyrene with the highest effect at 50 W in relation to hydrogen yield. The theoretical values for carbon dioxide yields were higher than experimental values, suggesting a negative synergistic effect. Raising the plasma power from 50 to 70 W increased the yields of the gases. As the input plasma power was increased, the electric field and the electron temperature will be increased, generating more energetic electrons, radicals, and other reactive species and leading to an increase in the breakdown of the volatile pyrolysis molecules and consequently higher gas yields.

The synergistic effects on the oil composition (mg/g of feedstock) for the main compounds produced during pyrolysis alone and the pyrolysis with nonthermal plasma processing of biomass and polystyrene at 50 and 70 W are exhibited in Figure 10c. The highest synergistic effect on oil composition can be seen for ethylbenzene, with the highest effect at the plasma input powers of 50 and 70 W, respectively. Styrene and 2,4-dimethyl-1-heptene concentrations in the product oil were negatively influenced by copyrolysis of biomass with the polystyrene. Styrene showed a marked negative synergistic effect under copyrolysis only conditions and also in the presence of the nonthermal plasma. Suriapparao et al.^58^ investigated the copyrolysis of different cellulosic biomasses and polystyrene (and also polypropylene) and also reported an increase in ethylbenzene and decrease in styrene for the copyrolysis of biomass and polystyrene. They suggested that as ethylbenzene is a hydrogenated product of styrene, the increase in yield was due to intermolecular hydrogen transfer between biomass and polystyrene radicals and intermediates. Methylglyoxal, which is an oxygenated compound, was produced at less than the expected amount at the plasma input powers of 50 and 70 W, indicating a deoxygenation effect with the nonthermal plasma.

It should be noted that all the experiments involved a first-stage pyrolysis; therefore, interaction of the biomass and polystyrene during the pyrolysis stage will involve some interaction processes. The biomass in this work consisted of newspaper, wood, and cardboard, which are composed of the biopolymers cellulose, hemicellulose, and lignin, each of which thermally decomposes at different temperatures. Cellulose thermally decomposes over the temperature range of 330–450 °C, hemicellulose over 200–330 °C, and lignin over a wider range of 250–550 °C.^59^ Polystyrene has a pyrolysis decomposition temperature range that covers the decomposition range of the cellulose and lignin biomass components of between 410 and 470 °C.^60^ The pyrolysis of polystyrene produces very high concentrations of styrene, with lower concentrations of benzene, toluene, alkylated benzenes, and naphthalene.^61^ Consequently, the oil produced from the mixed polystyrene and biomass will produce a highly aromatic product oil. For example, a product oil containing high concentrations of styrene, methylstyrene, toluene and ethylbenzene was produced from the copyrolysis of biomass and polystyrene.^42^ Reshad et al.^62^ used a semibatch pyrolysis reactor to study the copyrolysis of biomass and polystyrene and showed high concentrations of styrene, methylstyrene, and xylene, but aliphatic compounds in significant concentrations were also produced. Interaction of biomass and polystyrene as copyrolyzed feedstocks in a fixed-bed reactor at 500 °C was reported by Muneer et al.^57^ that resulted in a higher product oil yield than expected from processing of the individual feedstocks. The authors attributed the increase in oil yield to hydrogen donation from polystyrene to biomass decomposition. Interaction of mixed polystyrene and biomass has also been reported under thermal decomposition conditions using a thermogravimetric analyzer with a 40% deviation in the weight loss profiles compared to the individual samples.^63^ It is suggested that the radicals produced from the pyrolysis of the biomass biopolymers react with the polystyrene polymer, initiating polymer bond scission.^28^ Özsin and Pütün,^64^ investigated the synergistic interaction of polystyrene and biomass during copyrolysis using a thermogravimetric analyzer coupled to an infrared spectrometer and gas chromatograph/mass spectrometer. They reported a positive synergy, increase in liquid yield and negative synergy, and decrease in gas and char yields due to interaction. The synergetic effects were attributed to the role of polystyrene acting as a hydrogen donor leading to the generation of free radicals that initiate the inter-reaction between the polystyrene and the biomass.

The copyrolysis (no plasma) of the polystyrene and biomass mixture produced a marked reduction in styrene and increase in ethylbenzene in the product oil. Ethylbenzene has been reported as a hydrogenated product of styrene, and it has been suggested that intermolecular transfer of hydrogen between the polystyrene derived pyrolysis radicals and intermediates and the biomass leads to the production of ethylbenzene.^58^ Suriapparao et al.^58^ investigated the microwave copyrolysis of biomass and polystyrene. They reported that the presence of polystyrene increased the expected yield of aromatic hydrocarbons in the product oil. They also reported that the presence of the polystyrene in the copyrolysis with biomass considerably reduced the yield of phenolic compounds (phenols, guaiacols, and syringols) and other oxygenated groups (furan and cyclopentanone) in the product oil. They suggested that the interaction of intermediate species derived from the pyrolysis of polystyrene suppressed the formation and consequent yield of these oxygenated compounds due to the interaction with the biomass volatiles and improved the quality of the product oil.

In this work, the presence of the nonthermal plasma generates a reaction environment consisting of high-energy electrons, free radicals, and excited species that react with the biomass pyrolysis volatiles and will further enhance this interaction of the polystyrene derived intermediates with the biomass pyrolysis volatiles, further reducing the oxygen-containing compounds. However, the process is characterized by intricate chemistry, particularly in the context of bio-oil upgrading.^46^ In a DBD nonthermal plasma, the mean electron energy typically falls within the range of 1–10 eV. The Maxwellian electron energy distribution function suggests that as the average electron energy increases, a greater number of electrons with higher energy levels are generated.^65^ The energy in DBD plasma is high enough to break most chemical bonds, resulting in the production of ions, free radicals, excited molecules, and energetic electrons, among other active species.^45^ The order in which various chemical bonds break depends on the chemical binding energy. Illustrating the mechanisms or pathways for breaking down biomass and polystyrene volatiles in a plasma system poses significant challenges. However, some DBD plasma mechanisms for more simple feedstocks, such as tar model compounds, have already been suggested in the literature. Liu et al.^45^ proposed a mechanism for toluene reforming in a DBD nonthermal plasma reactor at 75W input plasma power. Toluene can be decomposed through either direct electron attack on toluene or reacting with active species, such as •OH, O•, O_3_, and N•. The sequence in which various chemical bonds are broken is determined by the chemical binding energy. The CH_3_ group on the benzene ring has a C–H binding energy of 3.7 eV, the aromatic ring has a C–H binding energy of 4.3 eV, the aromatic ring and methyl group have a C–C binding energy of 4.4 eV, the aromatic ring has a C–C binding energy of 5.0–5.3 eV, and the C=C binding energy is 5.5 eV.^45^ During the nonthermal plasma reaction, small activated radicals are generated by breaking C–H, C–O, and O–H bonds, each with lower mean energies than electron energies. Subsequently, new compounds can be formed through the recombination of these radicals. The methyl radicals, produced by breaking the O–CH_3_ or C–CH_3_ chemical bonds, may further undergo breakdown into CH_2_, CH, and H free radicals within the plasma reactor. Consequently, these methyl radicals can be fragmented *in situ* to generate H free radicals (CH_2_, CH, H).^27^ Nguyen et al.^38^ investigated the mechanism of high-density polyethylene (HDPE) decomposition in the presence of nonthermal plasma. The decomposition of HDPE under the plasma-only route occurs in a manner similar to that under the thermal-only route. As a result, long-chain volatiles released during HDPE decomposition generate carbocations through collisions with highly energetic species, such as electrons (excited electrons with energy in the range of 0–10 eV) and N_2_* (with an energy of 6.2 eV). This process reduces the energy barrier for the dissociation of C–H bonds (C–H = 4.3 eV) and C–C bonds (C–C = 3.4 eV), leading to an increased level of cracking of heavier hydrocarbons into gaseous products.

## 4 Conclusions

In this study, polystyrene plastic waste was coprocessed with waste biomass in a two-stage reactor to generate an improved and upgraded bio-oil. Initially, the pyrolysis of polystyrene and biomass produces volatile components, which then proceed to the second stage for cracking and autohydrogenation reactions under nonthermal plasma conditions. The findings suggest that nonthermal plasma facilitates *in situ* hydrogen generation, serving as a hydrogen source for the hydrogenation of biomass pyrolysis volatiles.

Increasing the input plasma power led to slight increases (∼3 wt %) in gas production for polystyrene, biomass, and the polystyrene–biomass blend, along with a corresponding decrease in liquid yield. There was a minor synergistic effect between biomass and polystyrene in terms of overall oil and gas yield with some indications of interaction in gas and oil composition.

For polystyrene processing alone, the addition of the nonthermal plasma resulted in higher yields of hydrogen, methane, and C_2_–C_4_ hydrocarbons, whereas the amount of monocyclic aromatic compounds increased and polycyclic aromatic compounds slightly decreased. Increasing the input plasma power from 50 to 70 W enhanced all these trends.

For biomass processing alone, the addition of the nonthermal plasma resulted in higher yields of carbon monoxide, carbon dioxide, hydrogen, and methane accompanied by a reduction in most oxygenated compound groups in the liquid phase. Again, as the input plasma power was increased, these effects were increased.

At an equal mass ratio of 1:1 biomass and polystyrene, the introduction of the nonthermal plasma led to higher yields of carbon monoxide, carbon dioxide, hydrogen, methane, and C_2_–C_4_ hydrocarbons in the gas phase. Moreover, as input plasma power was increased to 70 W, there were an increase in single-ring aromatics and a decrease in polycyclic aromatic compounds in the oil phase, with the presence of oxygenated compounds in the product oil reducing to almost zero at input plasma powers of 50 and 70 W. There was evidence of interaction of the volatiles derived from biomass and polystyrene in the copyrolysis process. It was suggested that this is due to the radicals and intermediates produced from the pyrolysis of polystyrene, which aid the decomposition of the biomass biopolymers. This process is enhanced in the nonthermal plasma environment due to the high-energy electrons that generate radicals and intermediates from the polystyrene volatiles that interact with the biomass volatiles, increasing the aromatic content and reducing the oxygenated compounds in the product oil.
